# Dengue Fever Associated with Acute Urticaria

**DOI:** 10.4269/ajtmh.24-0052

**Published:** 2024-06-04

**Authors:** Thanh Nguyen Huu, Thang Quoc Le, Thuy Tran Thi Phuong

**Affiliations:** ^1^ Department of Internal Medicine, College of Health Sciences, VinUniversity, Hanoi, Vietnam;; ^2^ Department of Infectious Diseases, Vinmec International Hospital, Hanoi, Vietnam

**Figure 1.**
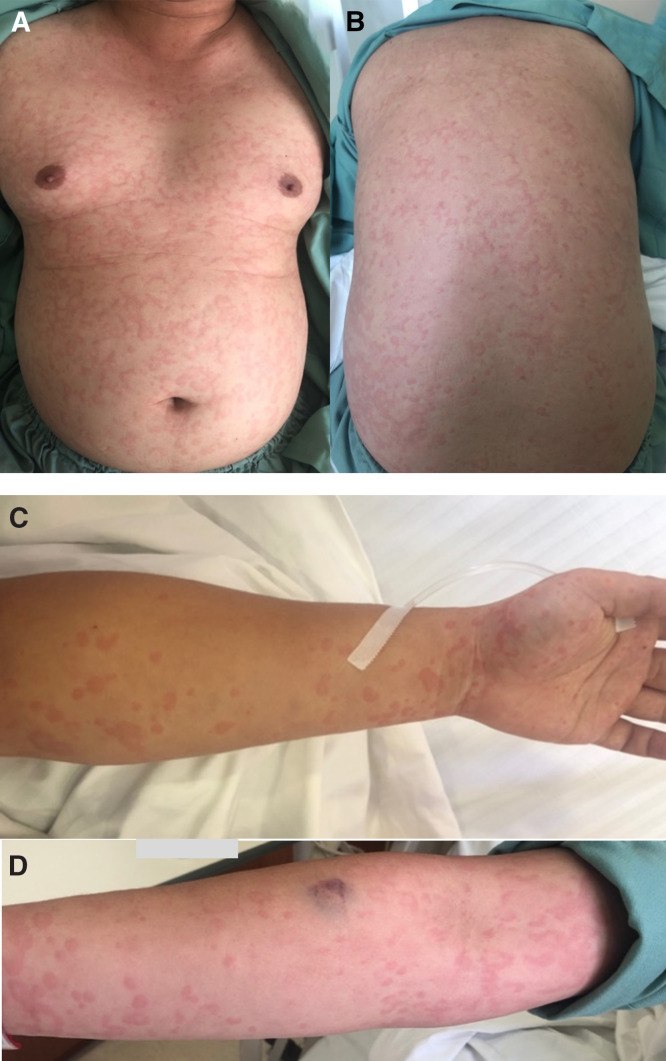
(**A** and **B**) Generalized erythematous, partially confluent wheals on the chest, abdomen, and back. (**C** and **D**) Generalized erythematous, partially confluent wheals on both arms.

A 25-year-old male in Hanoi, Vietnam, with a history of acetaminophen allergy, presented with fever, rash, and 6 hours of nonproductive cough. Over the subsequent 4 hours, a pruritic rash developed, starting in the trunk and then spreading to the arms and legs. This was the first time he had experienced this type of rash. He had not taken any new medications, including paracetamol, or new food. His temperature was 39°C; otherwise, his vital signs were normal. Partially confluent, erythematous wheals were present on the chest, abdomen, and arms (Figure 1) without angioedema. Laboratory tests revealed a normal white blood cell count of 5.3 × 10^9^/L, hematocrit of 47%, platelet count of 227 × 10^9^/L, normal liver enzymes, and C-reactive protein of 19.6 mg/dL (normal, <0.5 mg/dL). Testing for dengue fever was positive for the dengue virus nonstructural protein 1 antigen (OnSite^®^ Rapid Test, CTK Biotech, Poway, CA), establishing the diagnosis as dengue fever. He was treated with oral chlorpheniramine, methylprednisolone, and other supportive care. The rash mostly resolved within 24 hours and was gone by 3 days. He fully recovered and was discharged on day 7 of illness.

Rash is common in dengue fever, consisting of transient flushing erythema on the first and second days, followed by maculopapular or morbilliform skin findings and petechiae and rounded islands of sparing occurring after 3–5 days of the disease.^1^ Maculopapular rashes in infectious diseases are thought to be caused by adaptive immune responses against pathogens, commonly viruses, mediated by T-lymphocytes alone or both T-cell and B-cell responses that release the mediators, toxins, and antibodies.^2^ In dengue fever, it is hypothesized that the rash is due to inflammatory cell infiltration and dermal edema after increased vessel permeability.^3^

Urticaria occurs in the superficial dermis and is caused by vascular dilation and fluid leakage into the skin, triggered by histamine and vasodilator mediators released from mast cells and basophils.^4^ Infections can trigger urticaria. Dengue fever associated with acute urticaria is rarely reported. Recently, dengue viruses have been shown to infect the skin mast cells, leading to release of multiple mediators.^5^ Additional studies are required to investigate the association of dengue fever and urticaria, but this physical finding is important to understand more fully in this disease.
